# Leading Women in Respiratory Clinical Sciences: Letter From Hong Kong

**DOI:** 10.1111/resp.70013

**Published:** 2025-03-03

**Authors:** Janelle Yorke, Naomi Takemura

**Affiliations:** ^1^ Director of JC STEM Lab of Digital Oncology Care Enhancement (DOCE) Hong Kong SAR China; ^2^ The Hong Kong Polytechnic University, School of Nursing Hong Kong SAR China

**Keywords:** equality, leadership, nursing, respiratory, STEM

## Abstract

Special Series: Leading Women in Respiratory Clinical Sciences

*Series Editors: Anne‐Marie Russell and Kathleen O Lindell*

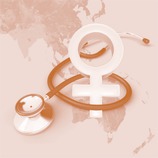

**See related** editorial

1

In Hong Kong, the field of respiratory clinical sciences is witnessing an increasing influence of women, particularly within the nursing profession, which is predominantly female. Nursing, deeply rooted in cultural values of caregiving and compassion, aligns with the traditional perception of women as natural caregivers within the Chinese context [1]. However, this cultural perception can also limit the recognition of nurses as leaders and researchers in clinical sciences. The societal expectation for Chinese women to prioritise family responsibilities often exacerbates this challenge, necessitating a delicate balance between professional aspirations and personal commitments. Despite these cultural barriers, Hong Kong, as a part of China and a former British colony, has undergone significant industrialisation and modernisation, leading to substantial economic and social development and notable improvements in women's status. The labour force participation rate for females increased from 47.9% in 1992 to 54.2% in 2021 [1].

Despite the rise in women's status, the traditional view of nursing as a supportive role rather than a leadership position in clinical sciences can hinder female nurses' opportunities to engage in clinical research. However, this perception is gradually shifting, as evidenced by recent developments. For example, Professor Yorke's recognition as the first and only nursing researcher to receive the Hong Kong SAR Global STEM Professorship and the only female Director of the Jockey Club (JC) STEM Lab at her institution, namely JC STEM Lab of Digital Oncology Care Enhancement (Figure 1). This achievement highlights the growing global recognition of nurses, particularly female nurses, in the academic and notably STEM field, where nursing is still emerging as a distinct discipline. Such awards not only celebrate individual accomplishments but also pave the way for greater inclusion and representation of women scientists in STEM professions worldwide. Thanks to the foresight of the HKSAR government and the JC, we have successfully established a nurse‐led STEM lab in Hong Kong, setting a precedent for future advancements.

**FIGURE 1:**
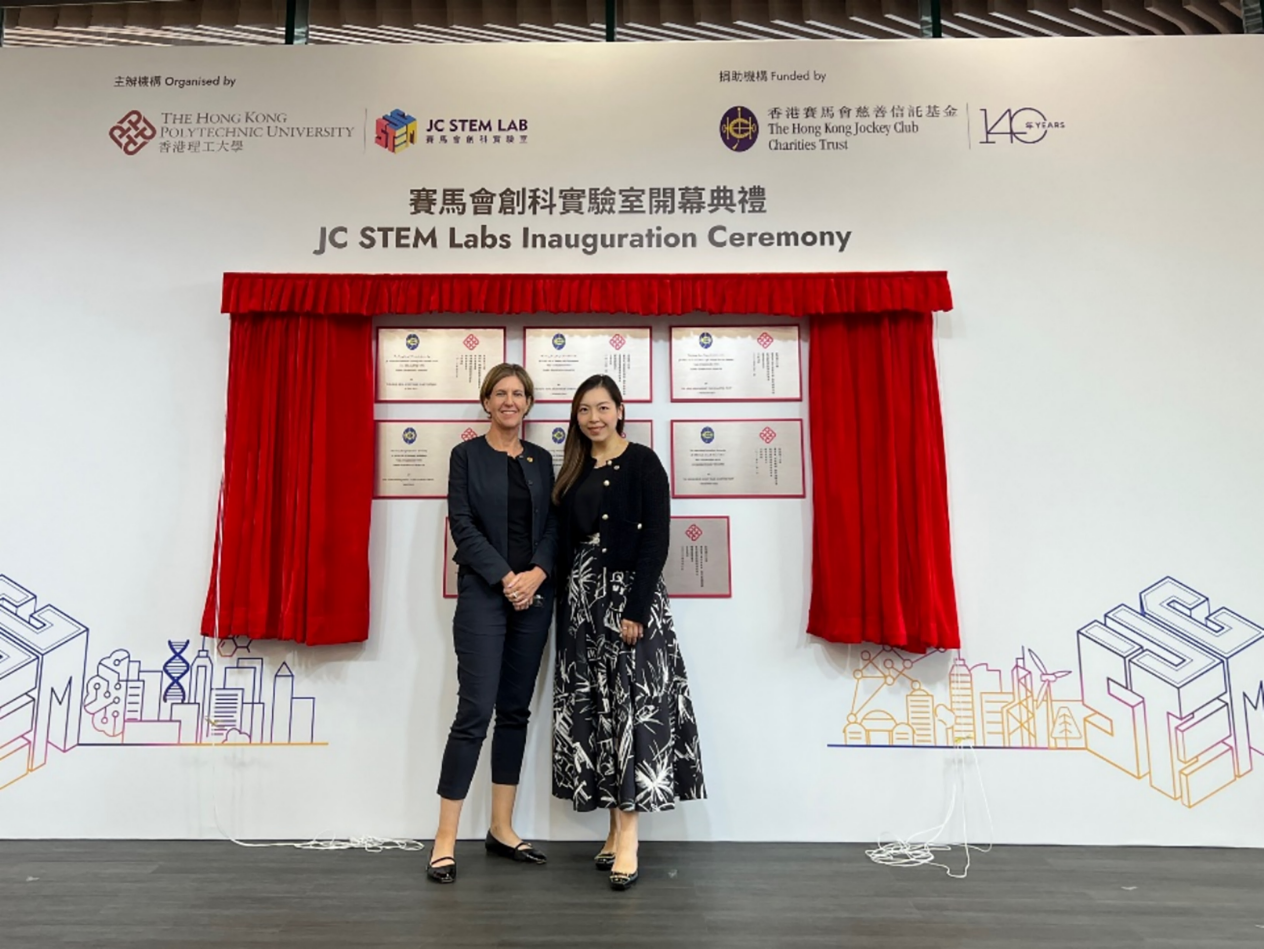
Prof. Yorke and Dr. Takemura at the inauguration of the Hong Kong Jockey Club STEM Lab: Digital Oncology Care Enhancement (DOCE), November 2024.

The reform of nursing education in Hong Kong, marked by the elevation of nursing education to degree level at tertiary institutions, has significantly enhanced the quality of healthcare services and strengthened the qualification of nurses [2]. This shift has also encouraged more nurses to pursue further studies beyond the Bachelor's degree, advancing to Master's and Ph.D. levels, thereby fostering a culture of continuous professional development and advanced practice. Consequently, nursing researchers in Hong Kong now possess greater experience and improved capabilities in conducting science‐based research, as evidenced by a significant increase in publications by Hong Kong nurse researchers since 2005 (*p* < 0.02) [3]. Furthermore, all three Hong Kong Government‐funded Schools of Nursing ranked within the top 40 for the Nursing subject listing of Quacquarelli Symonds (QS) Ranking, with PolyU Nursing ranked 31 in 2024. The QS World University Rankings, published annually, are a ranking of the world's top universities and subject areas including Nursing. The growing emphasis on evidence‐based practice in Hong Kong has motivated more nurses to pursue research opportunities. This shift has facilitated the creation of research‐focused roles within tertiary and healthcare institutions, such as nurses serving as professoriate staff in universities and as research nurses in healthcare settings, enabling nurses to actively contribute to and lead clinical research. For example, nurses are leading projects on patient‐centered care models, digital health technology integration, and chronic disease management. Additionally, the strong sense of community and collaboration within Hong Kong's healthcare system encourages interdisciplinary research. This collaborative spirit is a cultural strength that can be leveraged to promote the involvement of women, particularly nurses, in clinical sciences. Interdisciplinary research is often facilitated through structured mechanisms such as journal clubs and research seminars. These are organised by the Health Bureau, a policy bureau of the Government of Hong Kong, as well as by various institutions and non‐governmental organisations. Such initiatives provide platforms for professionals from diverse fields to exchange ideas and collaborate, thereby enriching the research landscape and promoting inclusivity within the healthcare sector.

While there are numerous opportunities and facilitators within Hong Kong healthcare workplaces, a cultural barrier remains due to the hierarchical structures that are prevalent in many institutions. In both academic and healthcare settings, decision‐making often adheres to a top‐down approach, which is traditionally more accepted within the Chinese context. This preference for authority and directives flowing from senior to junior staff can sometimes limit the autonomy of younger and less experienced nurses. The cultural emphasis on seniority and experience may inadvertently overshadow the contributions of junior staff. Furthermore, this dynamic can contribute to occupational stress and burnout among younger nurse academics, potentially leading to higher turnover rates [4]. To address this issue, it is essential to cultivate strong leadership and quality mentorship from senior nurse academics. Such measures are vital for retaining nursing faculty members and ensuring a supportive environment where all staff can flourish and contribute effectively [4].

The perspectives shared by experienced professionals in respiratory clinical sciences highlight the dynamic interplay between seasoned expertise and fresh perspectives. The commitment to nurturing and providing opportunities for junior faculty underscores the importance of succession planning. This approach emphasises the critical roles of mentorship, leadership, and collaboration in advancing the field. Such efforts are pivotal in elevating the representation and leadership of women in respiratory nursing science, contributing significantly to the global momentum in this vital area.

## Conflicts of Interest

The authors declare no conflicts of interest.

## Linked Content

This publication is linked to a related article. To view this article, visit https://doi.org/10.1111/resp.14841.
